# Supplementary material for: Heterozygote advantage cannot explain MHC diversity, but MHC diversity can explain heterozygote advantage
Source: bioRxiv. 2025 May 31:2025.05.27.656382. Preprint. [Version 1] doi: 10.1101/2025.05.27.656382 (PMC12154968; doi:10.1101/2025.05.27.656382)
Supplement: 1 [file NIHPP2025.05.27.656382V1-supplement-1.pdf]

bioRxiv preprint doi: https://doi.org/10.1101/2025.05.27.656382; this version posted May 31, 2025. The copyright holder for this preprint (which was not certified by peer review) is the author/funder. This article is a US Government work. It is not subject to copyright under 17 USC 105 and is also made available for use under a CC0 license.

Supplementary Material

Heterozygote advantage cannot explain MHC diversity, but MHC diversity can explain heterozygote advantage

Joshua L. Cherry

bioRxiv preprint doi: https://doi.org/10.1101/2025.05.27.656382; this version posted May 31, 2025. The copyright holder for this preprint (which was not certified by peer review) is the author/funder. This article is a US Government work. It is not subject to copyright under 17 USC 105 and is also made available for use under a CC0 license.

Supplementary Text S1

The Rate of Establishment of Expanded Haplotypes

In simulations of the symmetric Gaussian model with gene family expansion (Results), the long-term rate at which expanded haplotypes came to predominate, eliminating diversity, was about $2.68 \cdot 10^{-6}$/generation. This rate can be understood as follows. The average fitness of homozygotes for circulating single-gene haplotypes is only about 0.06, while most other genotypes have fitness close to 1. The selective advantage of an expanded haplotype, when rare, is therefore nearly equal to the rate of homozygosity, or about $1/n_e$, where n_e is the effective number of alleles. With only single-gene haplotypes, the harmonic mean of n_e is about 143 at equilibrium, so this selective advantage is about 0.00697. The fixation probability of an expanded haplotype is largely determined by its advantage when rare largely because fixation is almost guaranteed once it rises to a fairly low frequency. Thus, the fixation probability of a newly-arisen (single-copy) expanded haplotype is approximately $2s$, or about 0.0139. Such haplotypes arise at a rate of $2N \cdot 10^{-9}$, so fixations are predicted to occur at a rate of $2.79 \cdot 10^{-6}$/generation, very close to the observed rate of $2.68 \cdot 10^{-6}$.

bioRxiv preprint doi: https://doi.org/10.1101/2025.05.27.656382; this version posted May 31, 2025. The copyright holder for this preprint (which was not certified by peer review) is the author/funder. This article is a US Government work. It is not subject to copyright under 17 USC 105 and is also made available for use under a CC0 license.

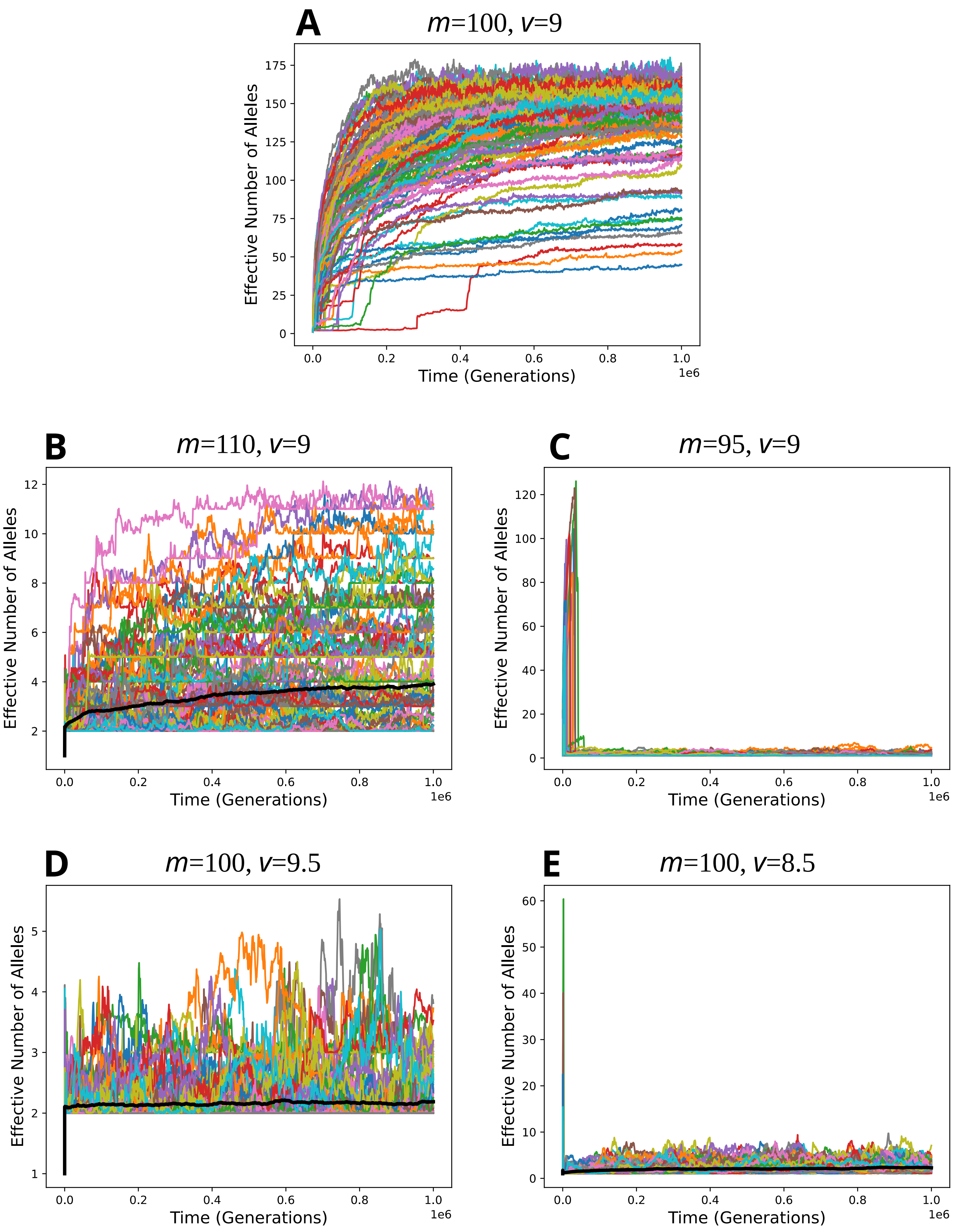

Fig. S1. Simulation results for the bitstring model with various values of parameters *m* and *v* and identical values of other parameters, including c_{max}. One hundred simulations runs are represented in each plot. Equilibrium diversity is high with *m*=100 and *v*=20 (**A**), but low if these parameters are changed slightly (**B-E**). The thick black curve in some of the plots (**B**, **D**, and **E**) represents the harmonic mean among runs.

bioRxiv preprint doi: https://doi.org/10.1101/2025.05.27.656382; this version posted May 31, 2025. The copyright holder for this preprint (which was not certified by peer review) is the author/funder. This article is a US Government work. It is not subject to copyright under 17 USC 105 and is also made available for use under a CC0 license.

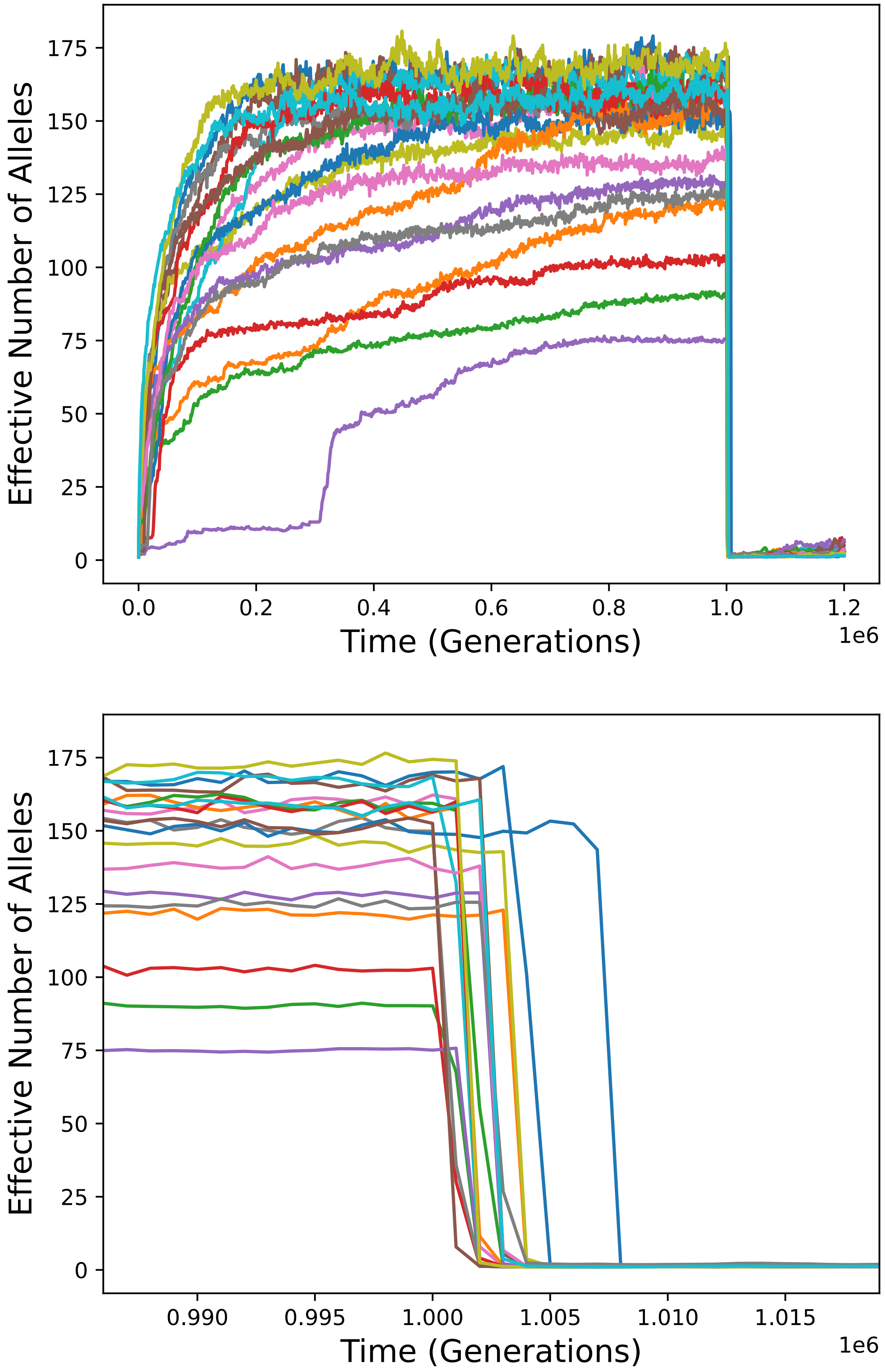

Fig. S2. Simulations like those in Fig. 6, top, except that diversity is allowed to accumulate for one million generations before mutations affecting the breadth of presentation are allowed. In all 20 runs, diversity collapses quickly once such mutations are allowed. The bottom panel is a horizontal zoom of the top panel.

bioRxiv preprint doi: https://doi.org/10.1101/2025.05.27.656382; this version posted May 31, 2025. The copyright holder for this preprint (which was not certified by peer review) is the author/funder. This article is a US Government work. It is not subject to copyright under 17 USC 105 and is also made available for use under a CC0 license.

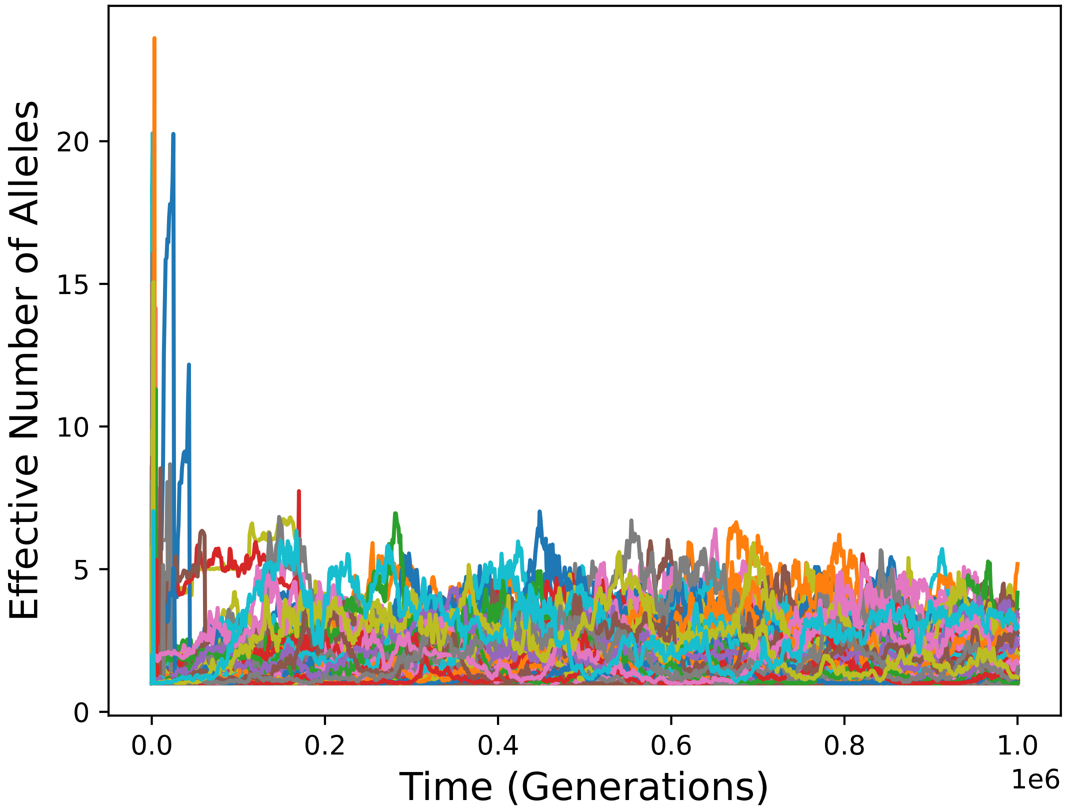

Fig. S3. Simulations incorporating a 50% decrease in all peptide detection probabilities in individuals with certain genotypes. Other conditions are as in Fig. 6, top. Despite the inclusion of this effect, alleles with higher presentation breadth quickly come to predominate, preventing the development of high diversity.
